# Comprehensive Secondary Structure Elucidation of Four Genera of the Family *Pospiviroidae*

**DOI:** 10.1371/journal.pone.0098655

**Published:** 2014-06-04

**Authors:** Tamara Giguère, Charith Raj Adkar-Purushothama, Jean-Pierre Perreault

**Affiliations:** Département de biochimie, Faculté de médecine et des sciences de la santé, Pavillon de recherche appliquée sur le cancer, Université de Sherbrooke, Sherbrooke, Québec, Canada; The Ohio State University, United States of America

## Abstract

Viroids are small, circular, single stranded RNA molecules that infect plants. Since they are non-coding, their structures play a critical role in their life cycles. To date, little effort has been spend on elucidating viroid structures in solution due to both the experimental difficulties and the time-consuming nature of the methodologies implicated. Recently, the technique of high-throughput selective 2′-hydroxyl acylation analyzed by primer extension (SHAPE) was adapted for the probing of the members of family *Avsunviroidae*, all of whom replicate in the chloroplast and demonstrate ribozyme activity. In the present work, twelve viroid species belonging to four different genera of the family *Pospiviroidae*, whose members are characterized by the presence of a central conserved region (CCR) and who replicate in nucleus of the host, were probed. Given that the structures of five distinct viroid species from the family *Pospiviroidae* have been previously reported, an overview of the different structural characteristics for all genera and the beginning of a manual classification of the different viroids based on their structural features are presented here.

## Introduction

In the diverse field of plant pathogens, viroids are extremely interesting because of both their small size (246–401 nucleotides; nt) and their single-stranded, non-coding RNA genome. Using only these characteristics, they are able to replicate autonomously and to move systemically in their host plants in which they induce visible symptoms of the viroid infection [1]. Since their discovery in 1971 [2], more than 30 species have been described and classified into two families: the *Avsunviroidae* and the *Pospiviroidae*. The members of the family *Avsunviroidae* replicate in the chloroplast through a symmetric rolling-circle mechanism that involves a self-cleaving hammerhead motif. In contrast, the members of the family *Pospiviroidae* replicate and accumulate in the nucleus via an asymmetric rolling-circle mechanism. Structurally, the members of the family *Pospiviroidae* are divided into five regions: the terminal left (TL), the pathogenic (P), the central conserved region (CCR), the variable (V) and the terminal right (TR). The *Pospiviroidae* are divided into three subfamilies, namely, *Pospiviroinae*, *Apscaviroinae*, and *Coleviroinae*. Further, the subfamilies are divided into genera based on sequence homologies in the CCR, the terminal conserved hairpin and the terminal conserved region [3]. Hence, the subfamily *Pospiviroinae* includes three genera, the *Pospiviroid*, the *Hostuviroid* and the *Cocadviroid*, whereas the subfamilies *Apscaviroinae* and *Coleviroinae* consists of one genus each, specifically the *Apscaviroid* and the *Coleviroid*, respectively.

All of the available data supports the notion that viroids do not code for any proteins. Hence, they are solely dependent on their structure for their replication, movement, pathogenesis and escaping from RNA interference (RNAi) mediated host defense [1]. Consequently, it is pivotal to elucidate viroids' secondary structures in solution in order to be able to understand their biological cycle. To date, the majority of the viroid structure representations present in the literature are based solely on computer-assisted predictions [4]–[15].

The modelling of the secondary structures of RNAs of the length of viroids has not always been a precise science. That said, new methods allowing for the incorporation of probing information obtained in solution into the structure predicting algorithm have yielded significantly more precise results. For example, the technique of selective 2′-hydroxyl acylation analyzed by primer extension (SHAPE) [16] coupled with computer based structure prediction using the RNAstructure software [17] has yielded good predictions for the structure of many RNAs [18], [19]. The SHAPE technique provides key information on the degree to which a nucleotide is paired. Nucleotides that are believed to be single-stranded react with a reagent creating a covalent adduct. When the primer extension reaction is performed, the reverse transcriptase stops one nucleotide prior to the adduct, thus creating a pattern of cDNAs of various lengths and quantities which is dependent upon the structure in question.

Previously, Dube *et al.* [20] predicted the structures of two variants of the *Peach latent mosaic viroid* (PLMVd) and compared them with those reported by Bussiere *et al.* [21]. In both cases the two structures were in good agreement, underscoring the efficacy of the SHAPE technique. The high-throughput SHAPE method, which requires the use of fluorescent-labelled probes and capillary electrophoresis to resolve the cDNA fragments produced after the primer extension step, can be use to assess the accessibility of each nucleotide. This simple, rapid, reproducible and non-radioactive approach can be performed in almost all laboratories without the need for any prior expertise [22]. Recently, it has been used to elucidate the structures of the RNA stands of both the (+) and the (−) polarities of all members of the family *Avsunviroidae*, as well as those of the grapevine hammerhead viroid-like, a circular RNA molecule that posseses a working hammerhead ribozyme in both polarities but has yet to be proven infectious [23]. In the present study, this redesigned SHAPE procedure was used to resolve the secondary structures of twelve viroid species from four genera of the family *Pospiviroidae*.

## Results and Discussion

### Probing viroids of the family *Pospiviroidae*

The goal of this study is to provide an overview of the secondary structures, in solution, of the viroids from the family *Pospiviroidae* by elucidating, at a one nucleotide resolution, the structures of many representative species by SHAPE. A previous study using traditional SHAPE coupled with computer assisted secondary structure prediction permitted the resolution of the structures of five distinct viroid species from three different genera of the family *Pospiviroidae*. More specifically, the structures of the *Citrus exocortis viroid* (CEVd), the *Citrus bent leaf viroid* (CBLVd), the *Hop stunt viroid* (HSVd), the *Citrus viroid-III* (CVd-III) and the *Citrus viroid-IV* (CVd-IV) were elucidated [24]. In order to achieve a global view of the structures adopted by the *Pospiviroidae* members, twelve other viroids from the four different genera were selected for SHAPE structure prediction (Table 1). This included five distinct species from the genus *Pospiviroid*, including the *Potato spindle tuber viroid* (PSTVd), one of the viroids widely used for biological studies, one representative member from the genus *Cocadviroid and* three representative members each from the genera *Apscaviroid* and *Coleviroid*.

**Table 1:** Viroids used in the present study for structure elucidation in solution.

Subfamily	Genus	Viroid	NCBI accession number	Size (nucleoties)	Starting sites	Reference
Pospiviroinae	Pospiviroids	PSTVd	AB623143	361	21, 174	[14]
		CSVd	V01107	356	94, 253	[5]
		TASVd	K00818	360	98, 175	[10]
		TCDVd	AF162131	357	172, 317	[11]
		CLVd	AY367350	373	181, 334	[15]
	Cocadviroids	CCCVd	J02050	247	3, 148	[6]
Apscaviroinae	Apscaviroids	ASSVd	M36646	330	1, 157	[7]
		PBCVd	D12823	315	109, 260	[8]
		CVd-OS	AB019508	330	1, 151	[9]
Coleviroinae	Coleviroids	CbVd-1	X52960	249	82, 193	[12]
		CbVd-2	X95365	301	108, 214	[13]
		CbVd-3	X95364	361	108, 262	[13]

### Secondary structures of the viroids from the genus *Pospiviroid*

#### Structure of PSTVd in solution

In general, the viroids of the genus *Pospiviroid* are well studied. The type species, PSTVd, was the first viroid to have its structure resolved in solution by enzymatic footprinting [25]. Initially, it was decided to reprobe PSTVd to demonstrate the potential of the hSHAPE methodology. For this purpose the PSTVd Dahlia variant, which is slightly different from the version probed previously in terms of nucleotide composition, was used. This PSTVd variant is interesting in that it was first isolated from the ornamental plant Dahlia, and it is known to induce mild symptoms in tomato plants [14].

**Figure 1:**
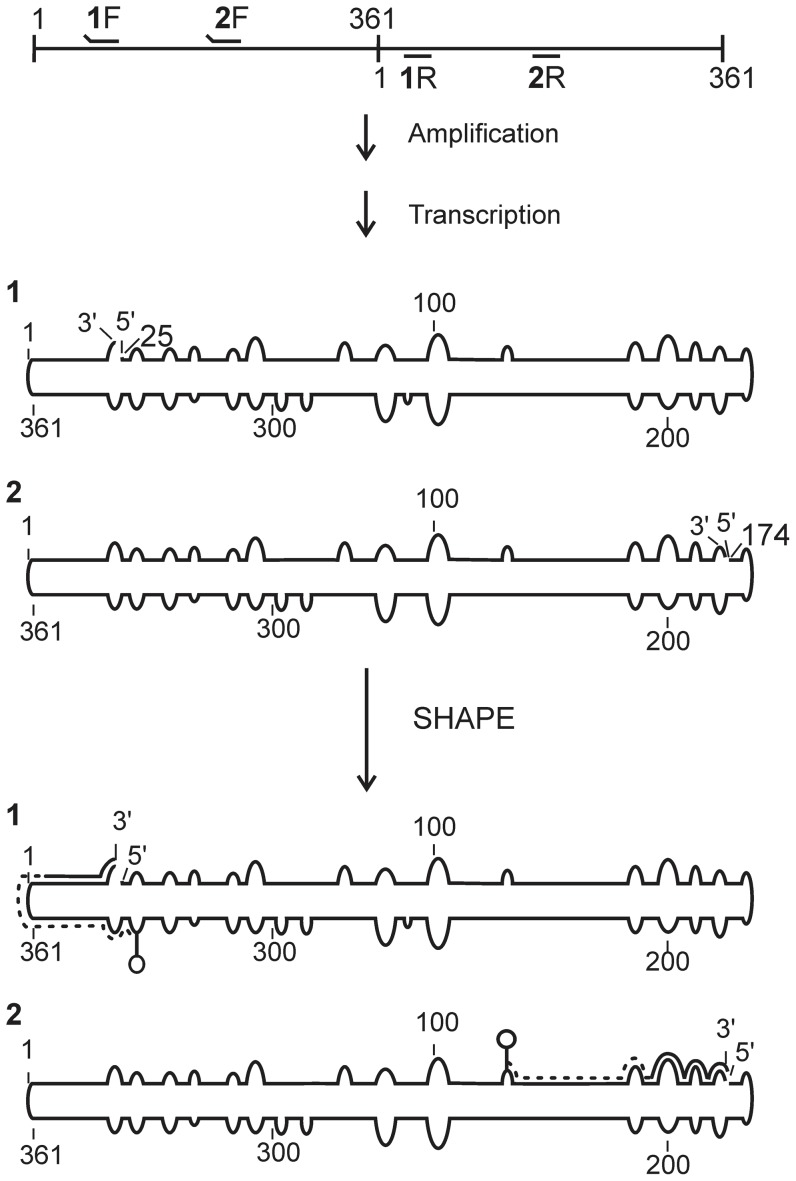
Schematic flow chart illustrating the detailed steps of the hSHAPE experiment with dimeric PSTVd. The numbered primers are used for the amplification of the PSTVd monomers (i.e. 1F/1R and 2F/2R). The raised ends of the primers represent the non-complementary regions, specifically the T7 RNA polymerase promoter, which is used for the run-off transcription. The RNAs obtained after amplification and transcription are numbered 1 and 2. These RNAs were subjected to the SHAPE reaction followed by primer extension. During the latter step the reverse transcriptase produces a cDNA fragment (dashed line) the length of the distance from the start of the RNA until the first adduct (e.g. circle on top of a line).

In order to produce the RNA required for the SHAPE probing, the head-to-tail dimeric copy of the viroid contained in a plasmid was amplified by PCR. Two different DNA templates of the complete monomeric viroid were prepared by using two sets of primers (Figure 1). The forward primers contained the T7 RNA polymerase promoter at their 5′-ends, and were designed such that the starting sites of the viroids possessed at least one guanosine residue for use in the subsequent *in vitro* transcription. To ensure the complete folding of the RNA molecules, the folding was performed at 37°C in the presence of magnesium chloride (MgCl_2_) prior to the addition of the SHAPE reagent. The SHAPE reagent reacts with the 2′-hydroxyl (2′-OH) of the ribose groups of a nucleotide when it is positioned in an accessible conformation, that is to say primarily when it is single-stranded. For this purpose, benzoyl cyanide (BzCN) was used because of its ability to react quickly with the RNA, as well as for its property of the rapid hydrolysis of any excess product in solution [26]. The variation between reactive and unreactive nucleotides was characterized by the early termination of the cDNA synthesis during the primer extension reaction that was catalyzed by a reverse transcriptase. Fluorescent-labeled primers were used for the primer extension reactions, thereby rendering the analysis of cDNAs up to 500 nt in length possible by capillary electrophoresis. This reaction mixture is hereafter referred to as SHAPE (+). In addition, a negative control without BzCN (SHAPE (−)) was also performed so as to monitor both the natural propensity of the reverse transcriptase to stop, and the regions where RNA degradation could have occurred. By using two monomeric viroids with different starting sites it was possible to obtain probing data on all of the nucleotides and to confirm the probing reactivity of each individual nucleotide. The resulting electropherograms were analyzed using the QuSHAPE software [27]. This software aligns, in semi-automatic fashion, the SHAPE (+) and the SHAPE (−) traces to both the sequencing reaction and the reference sequence of PSTVd. During the alignment process, it corrects the signal decay, integrates, normalizes and subtracts the data from the SHAPE (−) reaction from the SHAPE (+) reaction, producing the final SHAPE value. Each experiment was performed in duplicate for each RNA strand, resulting in four sets of SHAPE data for most nucleotides and two sets for both those nucleotides located at the primer binding sites and those located near this region. The resulting data represent the normalized and averaged reactivities of each nucleotide for all of the experiments. These data were used as soft thermodynamic constraints in the Fold tool of the RNAstructure software [17] to guide the RNA secondary structure prediction as described previously [23]. Briefly, non-reactive nucleotides are indicated by a value between 0 and 0.40. The reactive nucleotides are represented by values between 0.40 and 0.85, and the highly reactive nucleotides by values higher than 0.85. To evaluate the impact of the different starting sites, the levels of the SHAPE reactivities of each nucleotide from the different RNA strands were compared between each other. Nucleotides that had SHAPE reactivities that were either non-reactive (0–0.40) or highly reactive (>0.85) depending on the starting sites were considered as being ambiguous. Special attention was also taken when a few ambiguous nucleotides were found in the same region, as this could signify a change in the local structure.

**Figure 2:**
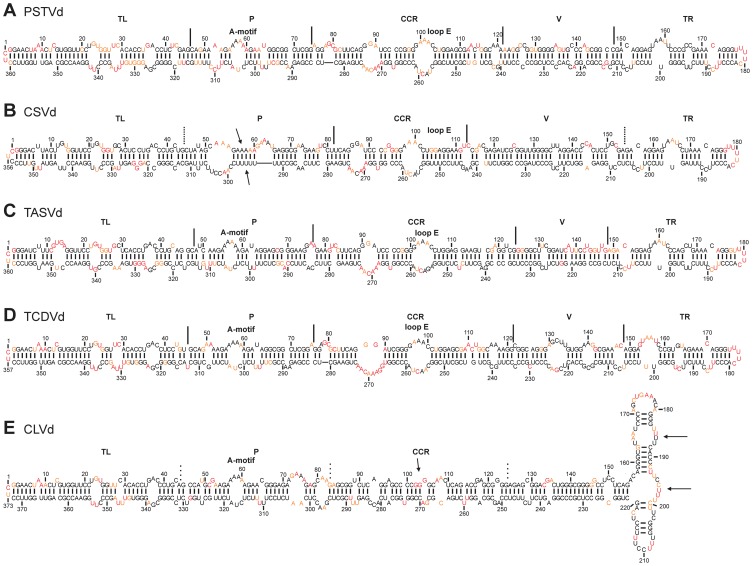
The most stable structures for the viroids from the genus *Pospiviroid*. The final structural models of PSTVd (A), CSVd (B), TASVd (C), TCVd (D) and, CLVd (E) obtained by SHAPE were folded with the RNAstructure software. The nucleotides in black denote those of low SHAPE reactivities (0–0.40). Those in orange are of intermediate reactivities (0.40–0.85) and those in red are highly reactive (>0.85). The different regions are either delimited (full lines), or approximated (dashed lines), and the presence of both the A-motif and the loop E are noted.

In the case of the probed PSTVd variant, the RNA strands started at either position 21 or 174. The oligonucleotides used for the primer extension were complementary to the sequence of the 3′ extremity of the RNA strands. The SHAPE reactivity profile for PSTVd was analyzed using QuSHAPE (Figure S1) and the values obtained were used in RNAstructure to obtain the final secondary structure (Figure 2A). The percentage of unambiguous nucleotides from the probings of both RNA strands was 94.46%, confirming that the starting site did not affect the folding of the RNA. Most of the reactive and the highly reactive nucleotides were located either in single-stranded regions, or in the adjacent positions. A 10.5% variation in nucleotide pairing was observed by comparing the structure predicted by SHAPE to that predicted without using the SHAPE values. Interestingly, when comparing the two most stable structures obtained with SHAPE, it was observed that the TL region might fold into two potential secondary structures. In the most stable one the residues from positions 327–333 are reactive, but the RNA structure prediction placed them in a double-stranded region. In the second most stable structure, however, two hairpins are formed between nucleotides 24–26 and 32–34 of the upper strand and nucleotides 327–331 and 337–342 of the lower strand. Since the stabilities of both structures are very similar (i.e. ΔG values of −267.0 kcal/mol and −262.0 kcal/mol), it is reasonable to propose that most likely the two structures are adopted in solution, thus explaining the SHAPE data. The presence of an A-motif in the P region composed of nucleotides 56–59 of the upper strand and 303–305 of the lower strand was observed. Additionally, loop E was formed at nucleotide positions 99–103 of the upper strand and 256–261 of the lower strand in the CCR of PSTVd. The overall profile of the reactivity obtained here, as well as the structural features described here, are similar to that obtained previously by enzymatic footprinting [25], confirming the accuracy of the SHAPE methodology.

#### Structures of four other *Pospiviroid*

After optimizing the SHAPE technique for rod-like viroids, four species from the genus *Pospiviroids* were selected based on several factors, including sequence identity. Neither the *Tomato planta macho viroid* (TPMVd), nor the *Mexican papita viroid* (MPVd), was probed because both possess a sequence identity of more than 85% with PSTVd. Moreover, their computer assisted predicted secondary structures were similar to that of PSTVd, suggesting, if the local variations are not considered, that all three viroids most likely adopt a similar structure in solution. The *Iresine viroid* (IrVd) was not probed because, firstly, little information was available, and, secondly, IrVd is not a threat to economically important plants like potato and tomato [28]. To date only three sequence variants of the *Citrus exocortis viroid* (CEVd; see Xu *et al.* [24]) have been probed. This left four members of the genus *Pospiviroid* to probe, namely the *Chrysanthemum stunt viroid* (CSVd), the *Tomato apical stunt viroid* (TASVd), the *Tomato chlorotic dwarf viroid* (TCDVd) and the *Columnea latent viroid* (CLVd).

The folding of CSVd, TASVd and TCDVd in solution produced structures very similar to that predicted for PSTVd. More specifically, the two probed RNA strands of CSVd were 96.1% unambiguous, and the variation caused by the probing, as compared to that of the structural model without probing, was 15.4%. The most important difference between CSVd and PSTVd is found in the structure of the A-motif that is located in the P region. Nucleotides 55–60 of the upper strand, which form the A-motif in PSTVd, are base-paired with the U-rich region comprised of nucleotides 293–297 of the lower strand in CSVd. However, this double-stranded region is surrounded by a loop to the left (nt 51–54 in the upper strand and 298–305 in the lower strand) and a bulge on the upper right (nt 61–65 in the upper strand), and is not simply included in a longer helical motif (Figure 2B). In the cases of TASVd and TCDVd, the probing of distinct RNA strands showed unambiguities of 97.2% and 96.6%, respectively (Figure 2C and D). In addition, they differed from their computer predicted structures by variations of 8.9% and 6.4%, respectively (Figure S1 and S2). Their structures are similar to that of PSTVd, with the exception that TCDVd possesses comparatively larger loops in both the V and the TR regions. Strikingly, all of these viroids, along with PSTVd, exhibited a conserved structure in the terminal of TR domain that consisted of either five (PSTVd and TASVd) or four (CSVd and TCDVd) loops.

The last member of the genus *Pospiviroid* to be probed was CLVd. Though CLVd can infect tomato, and is classified under the genus *Pospiviroid*, it is worth noting that it also shares some characteristics with *Hostuviroid* [15]. The two CLVd RNA strands had a SHAPE resolved structure with a percentage of unambiguous nucleotides of 97.9% (Figure S2), and the variation between the predicted and the final structural model was 17.4%. The structure of the TL region is very similar in all five viroids of this genus; however, a loop E was not detected in CLVd CCR. Additionally, a branched three-way junction was present in the TR region of CLVd (Figure 2E), implying the existence of some structural diversity within the genus *Pospiviroid*.

### Probing a viroid from the genus *Cocadviroid*

**Figure 3:**
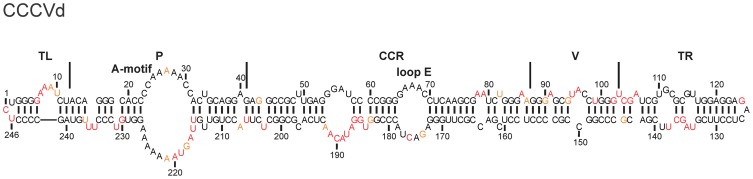
The most stable structure for a viroid from the genus *Cocadviroid*. The final structural model of CCCVd obtained by SHAPE and folded by the RNAstructure software. The nucleotides in black denote those of low SHAPE reactivities (0–0.40) Those in orange are of intermediate reactivities (0.40–0.85) and those in red are highly reactive (>0.85). The different regions are delimited by the full lines.

The genus *Cocadviroid* is composed of four species, the *Coconut cadang cadang viroid* (CCCVd), the *Coconut tinangaja viroid* (CTiVd), the *Hop latent viroid* (HLVd) and CVd-IV. Previously, structure of CVd-IV was elucidated using SHAPE [24]. In the present work, the structure of CCCVd, the type species of the genus, was elucidated. Unlike other viroids, CCCVd is known to possess two fast and two slow monomeric forms, (in terms of their electrophoretic mobilities) [29]. Here, one of the fast (246 nt) isomers was probed by SHAPE as it appears in the early stages of the disease and can induce severe symptoms in palms [29]. The probing results of the two distinct RNA strands were almost identical, with 98.0% of unambiguous nucleotides being obtained (Figure S3). The final structure revealed 16.7% variation in nucleotide pairings when it was compared to the computer predicted structure. The structure is characterized by the presence of a relatively large internal loop located in the P region (formed by the residues from nt 23–31 in the upper strand and nt 216–226 in the lower strand). This loop contains multiple adenosine residues, indicating the presence of a large A-motif in CCCVd. Moreover, the CCR of CCCVd contained a loop E structure, a feature characteristic of the genus *Pospiviroid* (Figure 3). A comparison of the structure of CCCVd with that of the previously probed CVd-IV [24] demonstrated the structural features of the genus, namely that both viroids exhibited similar structures in the CCR with both a loop E and an A-motif being present in the P region.

The sequence and the secondary structure of CTiVd exhibited homologies with those of CCCVd. Interestingly, both possess similar host ranges [30]. For this reason, the structure of CTiVd was not determined here. On the other hand, HLVd was not probed because the computer assisted structure of HLVd revealed a similarity with that of CCCVd presented in this report.

### Members of the genus *Apscaviroid* demonstrate structural diversity

The genus *Apscaviroid* consists of many members that infect a diverse set of hosts including apple, citrus, grapevine and pear. Previously, the structures of two viroids, the *Citrus III viroid* (CVd-III) and the *Citrus bent leaf viroid* (CBLVd), both of which infect citrus plants, were resolved using radiolabelled probes [24]. Therefore, the structures of other viroids which possess different host ranges were elucidated. For this purpose, initially, the structures of the *Apple scar skin viroid* (ASSVd), the *Apple dimple fruit viroid* (ADFVd), the *Grapevine yellow speckle viroids* -*1* and -*2*, (GYSVd-1 and -2), the *Australian grapevine viroid* (AGVd), the *Pear blister canker viroid* (PBCVd), the *Citrus viroid LSS* (CVd-LSS) and the *Citrus viroid-OS* (CVd-OS) were predicted using a computer-based algorithm. Interestingly, ASSVd, ADFVd, GYSVd-1, GYSVd-2 and AGVd folded into rod-like structures, whereas PBCVd and CVd-OS most stable structures revealed a simple branched and a highly branched structure (Figure S5), respectively. Therefore, ASSVd, PBCVd and CVd-OS were selected for elucidation of their secondary structures by SHAPE.

**Figure 4:**
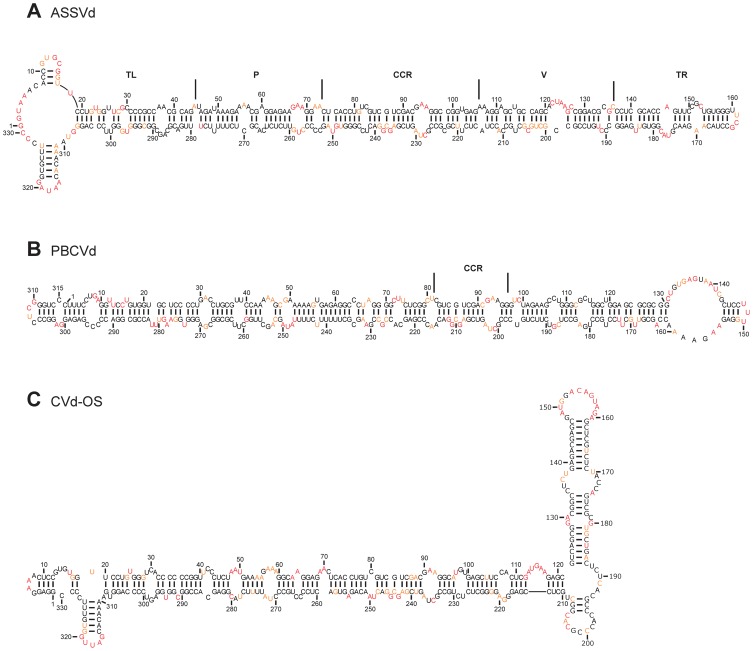
The most stable structures for the viroids from the genus *Apscaviroid*. The final structural models for ASSVd (A), PBCVd (B) and CVd-OS (C) obtained by SHAPE and folded by the RNAstructure software. The nucleotides in black denote those of low SHAPE reactivities (0–0.40). Those in orange are of intermediate reactivities (0.40–0.85) and those in red are highly reactive (>0.85). The CCR are delimited by the full lines.

Initially, ASSVd, the type species of the genus, was examined. It is 330 nt in length and causes severe skin scarring, dappling or cracking in the surface of the apple fruit [31]. The investigation of the structures of both RNA strands was unambiguous up to 97.6%. The most stable structure presented here possesses a branched structure in the TL region (Figure 4A). This prediction is in contrast to the result of the classical structure prediction algorithm, which uses mere sequence information and predicts a typical rod-like structure (Figure S5). The percentage of change between these two most stable predicted structures is 19.1%. Further, based on the SHAPE data, the most stable structure revealed a ΔG of −233.8 kcal/mol, and the second most stable, which displayed rod-like secondary structure, of −232.2 kcal/mol. The latter structure differs from the computer predicted structure by 12.5% (Figure S6).

In contrast to ASSVd, PBCVd, which showed a branched structure in *in silico* analysis, showed a classical rod-like structure when the SHAPE values were used (Figure 4B). A 46% variation in nucleotide pairing was noted between the two structures, mainly due to the branched secondary structure obtained in the TL and the P regions of the computer predicted structure (Figure S7). Interestingly, the two distinct RNA strands used for the probing in solution exhibited a high level of unambiguous nucleotides (99.4%).The final predicted structure in solution is characterized by a large internal loop located in the TR region (i.e. positions 131–143 and positions 155–161), the lower strand of which is A-rich.

The last member analyzed was CVd-OS. This is the one viroid that shares sequence similarity with both CVd-III and ADFVd in both the putative central and the TL regions [9]. Further, the sequences of the lower strands of the V and TR regions share high sequence similarities with CEVd [9]. The two RNA strands used for predicting the structure showed an excellent percentage of unambiguous nucleotides (96.7%). The structure obtained using the SHAPE probing data resulted in a central rod-like structure with branching on either side. More specifically, the TL and the TR regions each exhibited a three-way junction (Figure 4C). On the other hand, structure prediction without using the SHAPE data yielded a highly branched structure that included five stems located at the center of the structure (Figure S8). For this reason, structure prediction with and without SHAPE values resulted in a very high percentage of difference (59%). In fact these were the most divergent structures observed in this study.

Comparison of the structures of all of the viroids from the genus *Apscaviroid*, and more specifically of the three probed in this work, as well as those of CVd-III and CBLVd obtained from a previous report [24], revealed that the structures of the CCRs are very similar. It is worth noting that all of the members of the genus *Apscaviroid* share a similar sequence in the CCR. This region is structurally well conserved among the members of *Apscaviroid*, as is nicely illustrated by the three *Apscaviroid* members examined here (Figure 4). That said, the secondary structures predicted by the computer algorithm varied between the species as some demonstrated branched structures.

### Probing of the genus *Coleviroid*

**Figure 5:**
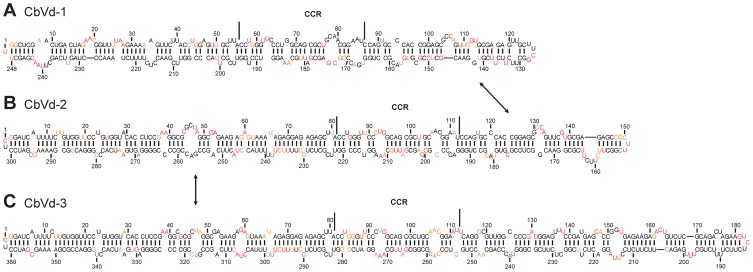
The most stable structures for the viroids from the genus *Coleviroid*. The final structural models for CbVd-1 (A), CbVd-2 (B) and CbVd-3 (C) obtained by SHAPE and folded by the RNAstructure software. The nucleotides in black denote those of low SHAPE reactivities (0–0.40). Those in orange are of intermediate reactivities (0.40–0.85) and those in red are highly reactive (>0.85). The CCR are delimited by the full lines. The arrows indicate the sequences that are identical in the three viroids.

All the viroids infecting *Coleus blumei* are grouped under the genus *Coleviroid*, which consists of six species: the *Coleus blumei viroid-1*, *-2*, *-3*, *-4*, *-5*, and *-6* (CbVd-1, -2, -3, -4, -5 and -6) [32], [33]. Recombination, a common phenomenon in CbVd, is observable in CbVd-2, CbVd-4 and CbVd-6 [13], [34], [35]. To our knowledge, none of the members of this genus had been probed previously. As a result, this study used three variants of CbVd for structure elucidation in solution, namely, CbVd-1 and CbVd-3, the natural variants and CbVd-2, a chimeric viroid obtained by the recombination of CbVd-1 and CbVd-3. The SHAPE procedure using two distinct RNA strands from all three species provided probing accuracies of 98.4%, 99.7% and 97.0%, respectively (Figure S9). When the sequences of the chimera parts are compared to those of the natural variants, the left side of the CCR of CbVd-2 was found to be almost identical to that of CbVd-3 while the right side was found to be almost identical to that of CbVd-1 [35]. In-depth analysis revealed that the reactivity of each nucleotide was similar when the identical domains of the three CbVd were compared (Figure 5). The final structures are all very similar and rod-like in nature with only a few minor structural differences being observed between the species. For example, a bulge was observed in the upper TR region CbVd-1 (nt 104–107), but was absent from the structure of CbVd-2. In this later variant, a bulge was present in the lower stem of the TR region (nt 159–163). Overall, the viroids from the genus *Coleviroid* displayed good structural homogeneity.

## Concluding Observations

This paper presents a large number of secondary structure elucidations of the family *Pospiviroidae's* members in solution. Structural precision at the single nucleotide level was achieved by using different starting sites to probe the viroids. The measure of the number of unambiguous nucleotides allowed for a thorough evaluation of the effect of the position of the starting sites. Analysis of the percentage of unambiguous nucleotide calculated, which ranged from 94.5% to 99.7%, indicated that the selected starting sites had negligible influences on the overall predicted structures in solution. This, conclusion, supports the notion that probing using this adapted SHAPE protocol is very accurate.

**Figure 6:**
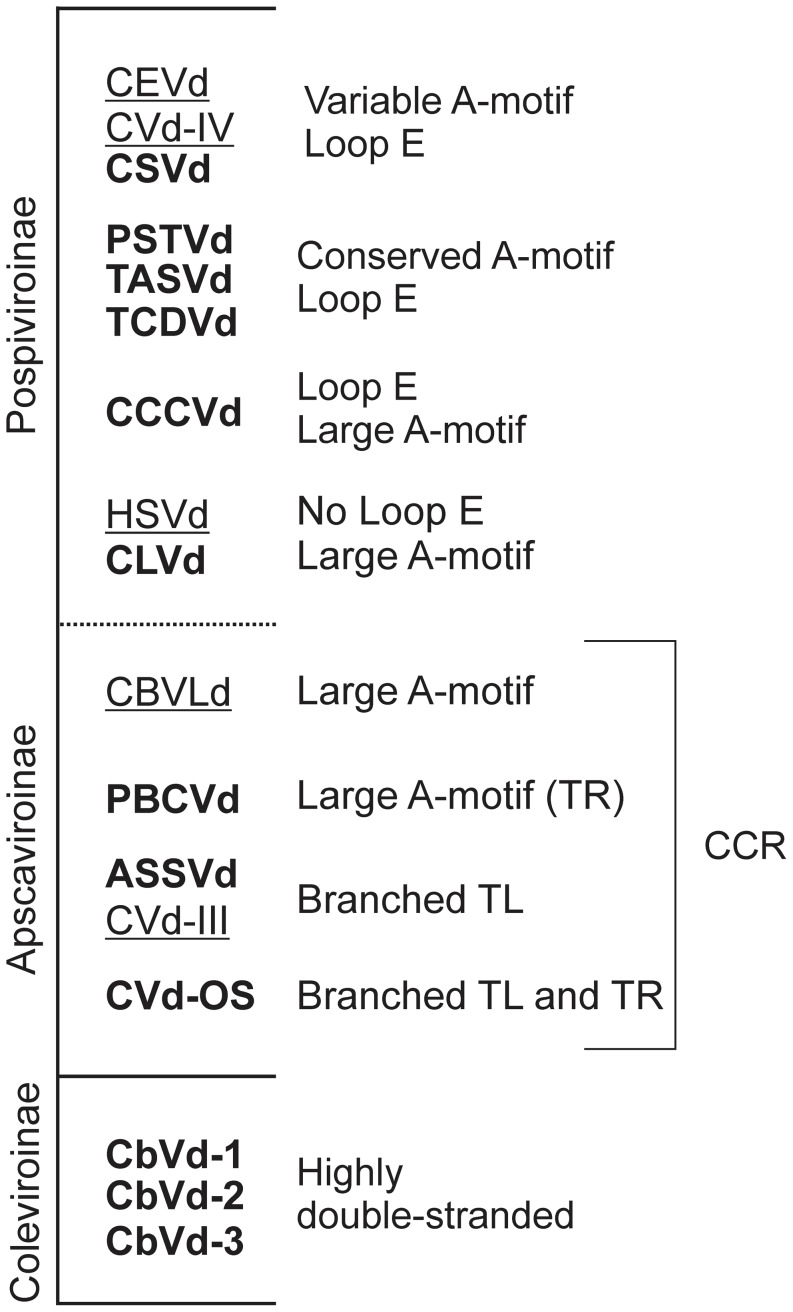
Index of the viroids with SHAPE elucidated structures, classified according to their characteristics. The viroids are also classified by their subfamilies. Viroids in bold are those reported in this article. The underlined names represent viroids whose structures were elucidated in other reports [24]. The characteristics described in the paper are listed on the right.

The structural comparison of the members of the various genera allowed the determination of distinct motifs in each genus (Figure 6). The viroids from the genus *Pospiviroid* probed in this report, as well as the three CEVd variants [24] previously probed, all have similar structures with the exception of CLVd. Typically, PSTVd, TASVd and TCDVd exhibited rod-like structures with an A-motif located in the P region and a loop E located in the CCR. All CEVd variants and CSVd had different A-motifs in their P regions, but all possessed a loop E. Conversely, CLVd lacked a loop E and showed a branched structure in the TR, but did demonstrate the presence of an A-motif in the P region. The lack of a loop E is one of the features present in HSVd, the only member of the genus *Hostuviroid* [24]. Verhoeven *et al*. [15] suggested that CLVd shares some characteristics with the genus *Pospiviroid*, as well as with the genus *Hostuviroid*. Biologically, CLVd is much more harmful to tomato than PSTVd and other members of the genus *Pospiviroid* are. This may be attributed to the branched structure of CLVd observed in this study. Furthermore, CLVd can infect cucumber [36], as HSVd can, but the other members of the genus *Pospiviroid* cannot, indicating that CLVd is quite different from the members of the genus *Pospiviroid* with respect to both its structural and its biological features.

CCCVd, of the genus *Cocadviroid*, exhibited structural similarity with the members of the genus *Pospiviroid*, with both possessing two loops in their CCRs and a loop with a conserved GGA sequence in the upper strand followed by a loop E. Additionally CCCVd also revealed the presence of an A-motif in the P region that is larger than those of the members of the genus *Pospiviroid*. Both of these features were also present in CVd-IV, another member of the genus *Cocadviroid* whose structure was elucidated previously [24]. Taking into account all three of these genera, and of the subfamily *Pospiviroinae*, the structures of eight species were elucidated. Detailed analysis clearly revealed that most of the *Pospiviroid* (except for CLVd) and the *Cocadviroid* have two loops in their CCR. HSVd did not reveal the presence of any of these loops in its CCR, indicating that the genus *Cocadviroid* is structurally closer to the genus *Pospiviroid* than to the genus *Hostuviroid*. On the other hand, an A-motif in the form of either a small or a large loop was present in all of the species except for CSVd and CEVd, which belong to the subfamily *Pospiviroinae*.

In the case of *Apscaviroid*, all three species probed here exhibited very different structures in solution, except for their CCRs which were identical in all of the members examined here. Some possible explanations for these differences are that all three of the probed viroids have different host ranges. Unlike the subfamily *Pospiviroinae*, all of the members of the subfamily *Apscaviroinae* are placed in only one genus, namely *Apscaviroid*, indicating the high possibility of structural diversity within this genus. Moreover, ASSVd demonstrated only 52.3% sequence identity to PBCVd, but is 61.7% homologous to CVd-OS. PBCVd and CVd-OS exhibited 48.7% sequence similarity. Two species of this genus, CVd-III and CBLVd, have been probed previously [24]. Both had similar CCRs, as was observed here, but differed in the rest of their structures, underscoring the high structural diversity within the genus *Apscaviroid*. Further, it is interesting to note that both CVd-III and CVd-OS exhibited a similar A-motif located in the P region, a feature that was not observed in the other members of the genus. At the same time, another citrus infecting viroid, CEVd of the genus *Pospiviroid*, also exhibited this type of A-motif. This indicates the need to regroup viroids by combining both their structural and their biological features.

Members of the genus *Coleviroid* do not possess any structural resemblance with the other genera except for their rod-like appearance. For example, their structures have neither an A-motif, nor a loop E. The viroids of this genus have percentages of base paired nucleotides of 70% and 74% for CbVd-1 and CbVd-2/3, respectively, which is higher than the viroids from the other genera. In summary, the different characteristics observed in the probing of viroids can help in determining the genus of a viroid. A tentative representation is shown in Figure 6.

It is important to remember that the SHAPE values help in the prediction of the RNA structure by suggesting which nucleotides should be single stranded and which ones are paired, but not with which nucleotide the pairing occurs. The prediction software is what takes into account the SHAPE data and generates a possibility for the most stable structure. In solution, it is possible that the RNA can adopt structures different than the proposed one. For this reason, the RNAstructure software proposes an ensemble of structures. Here, the most stable ones are presented for each viroid because they are the ones that are in best accordance with the SHAPE data. However, the RNA is a flexible molecule and some particularities were observed for both PSTVd and ASSVd in the second most stable structures that suggested that the alternative structures could also be found in solution. Importantly, alternative structures for the other probed viroids could also exist. Another consideration is the fact that this study is in solution, therefore the structure of these viroids *in vivo* could be different depending of the cellular environment.

In conclusion, a total of twelve new viroid structures were elucidated in solution. In addition, computer assisted predicted structures are also presented. One variant from at least one species from each genus has had its structure elucidated, including the type species for each genus. Prior to this report, simple computer based structure predictions suggested that all the members of the family *Pospiviroidae* were rod-like. That said, the structures predicted in solution clearly illustrated the diversity among the members of this family. Additionally, the data presented here clearly underlines the need to consider viroids as structural RNAs that possess individually different characteristics that permit a greater precision in their classification then currently exists.

## Materials and Methods

### Viroid origins

Plasmids containing head-to-tail dimers of the different viroids studied in this report were obtained from commercial synthesizers. Specifically, the constructs containing the head-to-tail dimers of CCCVd, ASSVd, CVd-OS, CbVd-1, CbVd-2 and CbVd-3 were purchased from BioBasics. The other constructs, CLVd, CSVd, TASVd and TCDVd, were ordered from Life Technologies (GeneArt gene synthesis platform). Finally, the PSTVd head-to-tail dimers were constructed by using monomeric PSTVd obtained from gBlock Gene Fragments (Integrated DNA Technologies, Inc). Briefly, a PSTVd DNA molecule containing *Bam*HI restriction enzyme site on either sides (*Bam*HI site is present in PSTVd at nt 87 to nt 93) was amplified by PCR and cloned in pGEM-T easy vector (Promega, Madison, USA). Plasmid containing PSTVd molecule was then digested with *Bam*HI. The DNA fragments were separated by electrophoresis on an agarose gel and purified by using QIAquick gel extraction kit according to the manufacturer's instruction (Qiagen). Thus purified PSTVd molecules were allowed to multimerize in the presence of T4 DNA ligase for 60 min at room temperature. To this, 1 µl of the *Bam*HI digested pBlueScript SK (+) vector (Stratagene) was added and vector-insert ligation was facilitated by prolonging the incubation for 30 min at room. Ligated products were transformed into competent *E. coli* cells. Selections of head-to-tail dimers were done by colony PCR and sequencing.

### Preparation of DNA templates

Double-stranded DNA of monomeric viroids was prepared by amplification from each DNA plasmid containing the viroid head-to-tail dimers. The amplifications were performed using purified *Pfu* DNA polymerase and a pair of oligonucleotides (Table S1). All of the forward primers included the T7 RNA polymerase promoter for the subsequent production of monomeric viroid RNA. For the PCR amplification of the monomeric viroid, an initial 1 min denaturation at 94°C was followed by 35 cycles of 1 min at 94°C, 1 min at 60°C and 1 min at 72°C in buffer containing 20 mM Tris-HCl pH 8.8, 10 mM (NH_4_)_2_SO_4_, 10 mM KCl, 0.1% Triton X-100, 20 mM dNTPs, 200 mM MgSO_4_, 200 µM of each primer and 2 µL of purified *Pfu* DNA polymerase. After the PCR amplification, the samples were heated at 72°C for 5 min, ethanol precipitated and a fraction of the resulting solution was analyzed on a 1% agarose gel.

### Preparation of RNA strands

In order to produce the viroid transcripts the DNA templates were incubated in the presence of T7 RNA polymerase in a buffer containing 80 mM HEPES-KOH (pH 7.5), 24 mM MgCl_2_, 2 mM spermidine, 40 mM DTT, 5 mM of each NTP, 0.004 units/mL pyrophosphatase (Roche Diagnostics), 40 units of RNAseOUT (Life Technologies) and 2 µL of purified T7 RNA polymerase. The resulting mixtures were incubated at 37°C for 90 min. Then, DNase RQ1 (2 µL) was added and the sample incubated for 20 min at 37°C. Two volumes of denaturing buffer (0.03% wt/vol each of bromophenol blue and xylene cyanol, 10 mM EDTA pH 7.5 and 97.5% vol/vol deionized formamide) were added prior to the purification of the RNA by denaturing gel electrophoresis (5% acrylamide and 8 M urea). The gels were visualized under UV light and the bands corresponding to the full-length monomeric viroids were excised and eluted (500 mM NH_4_OAc, 10 mM EDTA and 0.1% sodium dodecylsulphate (SDS)) overnight at room temperature. The RNAs were then ethanol precipitated and the pellets were washed with 70% ethanol prior to being dissolved in 0.5 X TE buffer (1X stock: 10 mM Tris-HCl (pH 7.5), 1 mM EDTA). The final concentration and purity of the RNAs were assessed by spectrometry.

### SHAPE reaction

In order, to perform the SHAPE reactions, RNA (5 pmol) was dissolved TE 0.5X buffer (8 µL). The samples were heated at 95°C for 3 min and then snap-cooled on ice for 5 min. The folding buffer (1 µL; 500 mM Tris-HCl pH 7.5, 500 mM NaCl) was then added and the sample incubated at 37°C for 5 min. To ensure complete folding of the RNA, 100 mM MgCl_2_ (1 µL) was added and the mixtures were kept at 37°C for another 30 min. Then, 600 mM of BzCN (1 µl) dissolved in DMSO was added to the (+) SHAPE reactions and DMSO (1 µL) alone to the (−) SHAPE reactions prior to ethanol precipitation in presence of glycogen (1 µL). The resulting pellets were washed with 70% ethanol and then were dissolved of TE 0.5X buffer (10 µL).

### Primer extension

The mixtures resulting from the SHAPE reactions were heated at 95°C for 2 min and then were snap-cooled on ice for 5 min prior to the addition of a 5′-fluorescently (VIC) labelled primer (1 pmol) complementary to the 3′ end of the RNA. The primers were annealed during a cycle of 5 min at 65°C, 5 min at 37°C and 1 min at 4°C before the addition of the primer extension buffer (4 µL 5X first strand buffer (Life Technologies), 1 µL of DTT (100 mM), 1 µL of dNTPs (10 mM) and 2 µL of DMSO). The reactions were heated to 52°C for 1 min and Superscript III (0.7 µL, Life Technologies) was then added and the reaction incubated at 37°C for 30 min. After completion of the primer extension reactions, the RNA was degraded with by the addition of 2 M NaOH (1 µL) and then heating for 5 min at 95°C. The cDNA was then ethanol precipitated and the pellets washed twice with 70% ethanol. The primer extension protocol was used to prepare a cDNA ladder for the capillary electrophoresis. This ladder was used as a sequencing reaction to help align the reactivity of each nucleotide with the sequence of the probed viroids. The main differences between the SHAPE reactions and the ladders were the use of another fluorophore (NED) on the primer extension oligonucleotides and of a concentration of either 0.75 mM or 1.5 mM of ddCTP for the reverse transcriptase reaction.

### Analysis by capillary electrophoresis

The cDNAs pellets containing either the SHAPE (+), the SHAPE (−) or the ladder reaction were sent to a genotyping facility for analysis on an ABI 3100 Genetic Analyzer. The samples were prepared as followed. Firstly, they were diluted in H_2_O (10 µL) and then formamide (10 µL) containing a Lyz labelled DNA ladder were added. An equal volume of diluted sequencing reaction was also added to the SHAPE reactions. The mixtures were then loaded into separate capillaries and the fluorescence measured.

### Analysis and viroid structures

The resulting electropherograms were analyzed by the QuSHAPE software using the defaults parameters [27]. The normalized reactivity values were averaged and loaded into the RNAstructure 5.5 software Fold tool as pseudo-energy constraints with a slope value of 1.8 kcal/mol and an intercept value of −0.6 kcal/mol as parameters. The structures with the lowest Gibbs free energies are presented here.

## Supporting Information

Figure S1 **The normalized SHAPE reactivities for the nucleotides of PSTVd, CSVd and TASVd.** The results are presented as a function of nucleotide position.
(TIF)

Figure S2 **The normalized SHAPE reactivities for the nucleotides of TCDVd and CLVd.** The results are presented as a function of nucleotide position.
(TIF)

Figure S3 **The normalized SHAPE reactivities for the nucleotides of CCCVd.** The results are presented as a function of nucleotide position.
(TIF)

Figure S4 **The normalized SHAPE reactivities for the nucleotides of ASSVd, PBCVd and CVd-OS.** The results are presented as a function of nucleotide position.
(TIF)

Figure S5 **The most stable structure obtained without SHAPE for ASSVd.** The secondary structure for ASSVd shown is the one predicted by the RNAstructure program.
(TIF)

Figure S6 **The second most stable structure of ASSVd obtained by SHAPE and folded by RNAstructure.** The nucleotides in black denote low SHAPE reactivities (0–0.40), those in orange are of intermediate reactivities (0.40–0.85) and those in red are highly reactive (>0.85). The different regions are delimited by the full lines.
(TIF)

Figure S7 **The most stable structures obtained without SHAPE for PBCVd.** The secondary structure for PBCVd shown is the one predicted by the RNAstructure program.
(TIF)

Figure S8 **The most stable structures obtained without SHAPE for CVd-OS.** The secondary structure for CVd-OS shown is the one predicted by the RNAstructure program.
(TIF)

Figure S9 **The normalized SHAPE reactivities for the nucleotides of CbVb-1, CbVd-2 and CbVd-3.** The results are presented as a function of nucleotide position.
(TIF)

Table S1 **Oligonucleotides used in the present work.** The T7 RNA polymerase promoter is denoted by the underlined sequences.
(DOC)
