# Supplementary figures and images for: Comprehensive Secondary Structure Elucidation of Four Genera of the Family Pospiviroidae
Source: PLoS One. 2014 Jun 4;9(6):e98655. doi: 10.1371/journal.pone.0098655 (PMC4045682; doi:10.1371/journal.pone.0098655)

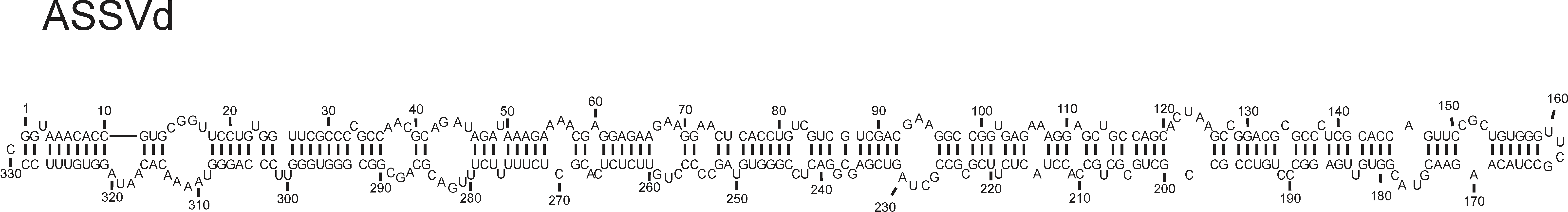

Supplement: Figure S5 — The most stable structure obtained without SHAPE for ASSVd. The secondary structure for ASSVd shown is the one predicted by the RNAstructure program. (TIF) [file pone.0098655.s005.tif]

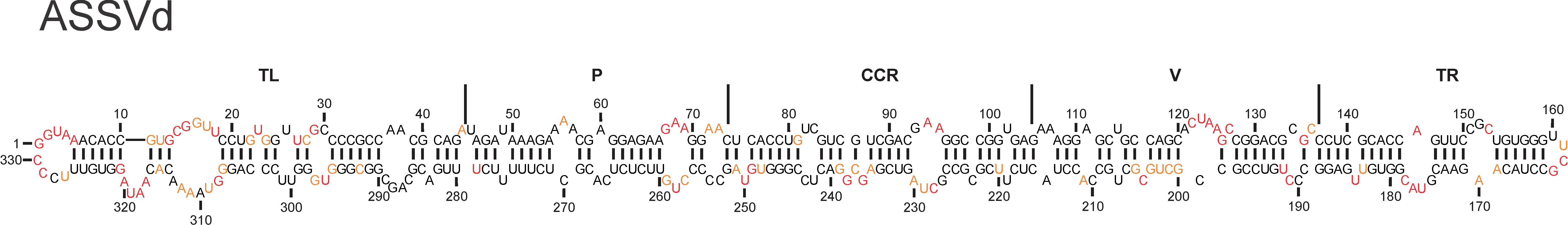

Supplement: Figure S6 — The second most stable structure of ASSVd obtained by SHAPE and folded by RNAstructure. The nucleotides in black denote low SHAPE reactivities (0–0.40), those in orange are of intermediate reactivities (0.40–0.85) and those in red are highly reactive (>0.85). The different regions are delimited by the full lines. (TIF) [file pone.0098655.s006.tif]

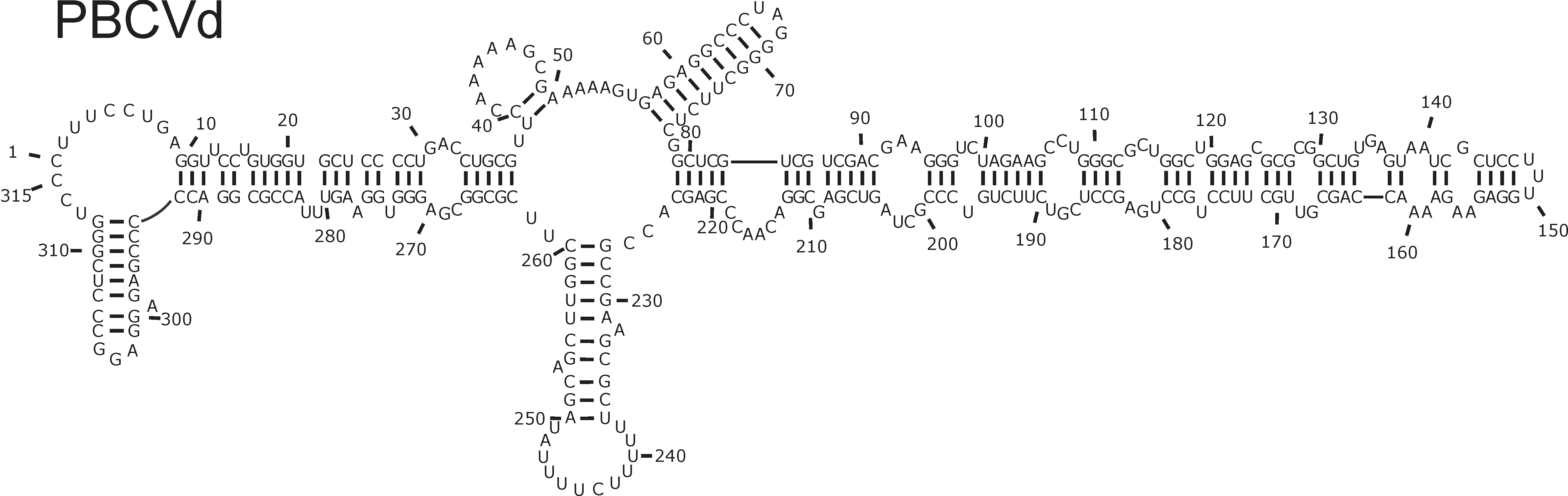

Supplement: Figure S7 — The most stable structures obtained without SHAPE for PBCVd. The secondary structure for PBCVd shown is the one predicted by the RNAstructure program. (TIF) [file pone.0098655.s007.tif]

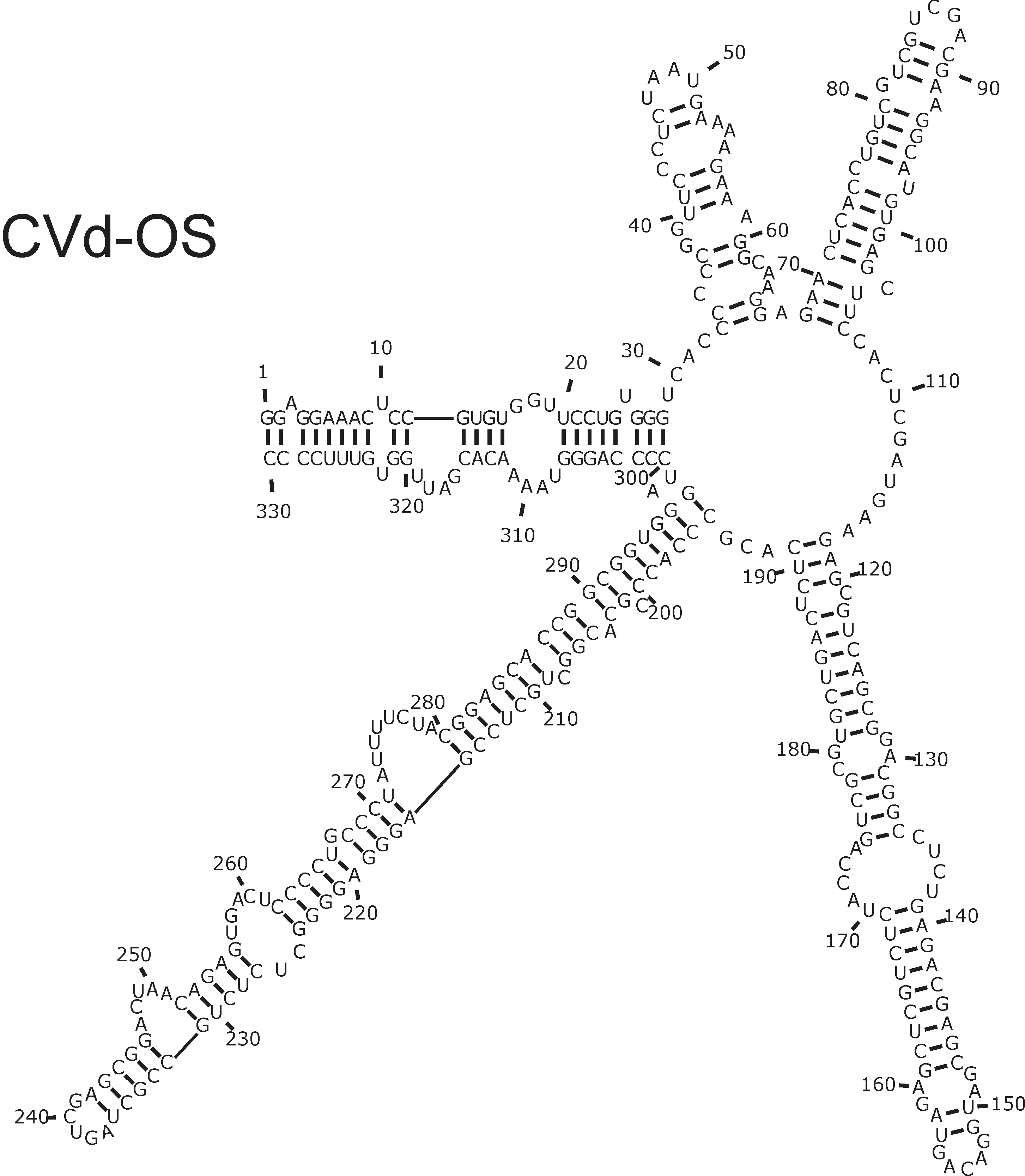

Supplement: Figure S8 — The most stable structures obtained without SHAPE for CVd-OS. The secondary structure for CVd-OS shown is the one predicted by the RNAstructure program. (TIF) [file pone.0098655.s008.tif]
