# Supplementary material for: Comprehensive Secondary Structure Elucidation of Four Genera of the Family Pospiviroidae
Source: PLoS One. 2014 Jun 4;9(6):e98655. doi: 10.1371/journal.pone.0098655 (PMC4045682; doi:10.1371/journal.pone.0098655)
Supplement: Table S1 — Oligonucleotides used in the present work. The T7 RNA polymerase promoter is denoted by the underlined sequences. (DOC) [file pone.0098655.s010.doc]

**Supplementary Table 1**. Information on the viroids characterized

| **Viroid** | **Starting site** | **Primer Name** | **Primer Sequence (5’-3’)** |
| --- | --- | --- | --- |
| PSTVd | 21 | T7-PSTVd-F3 | TAATACGACTCACTATACTGTGGTTCACACCTGACCT |
|  |  | PSTVd R3 | GAACCACGAGTTTAGTTCCG |
|  | 174 | T7-PSTVd-F2 | TAATACGACTCACTATAGGGTTTTCACCCTTCCTTTC |
|  |  | PSTVd R2 | TGTTTCGGCGGGAATTACTC |
| CSVd | 94 | T7_CSVd_F1 | TAATACGACTCACTATAGGGGAAACCTGGAGGAAGTC |
|  |  | CSVd_R1 | GGGGATCCCTGAAGGACTTC |
|  | 253 | T7_CSVd_F2 | TAATACGACTCACTATAGGCTACTACCCGGTGGAAAC |
|  |  | CSVd_R2 | AAAGGAAGCTTCGAAGACCG |
| TASVd | 98 | T7_TASVd_F1 | TAATACGACTCACTATAGGGGAAACCTGGAGGAAGTC |
|  |  | TASVd_R1 | GGGGATCCCTGAAGGACTTC |
|  | 175 | T7_TASVd_F2 | TAATACGACTCACTATAGGGTTTTCACCCTTCCTTTC |
|  |  | TASVd_R2 | TGTTTCAGCTGGGATTACTC |
| TCDVd | 172 | T7_TCDVd_F1 | TAATACGACTCACTATAGGGTTTTCACCCTTCCTTTC |
|  |  | TCDVd_R1 |  |
|  | 317 | T7_TCDVd_F2 | TAATACGACTCACTATAGGGGCGAGGGTGTTTAGCCCT |
|  |  | TCDVd_R2 | GGTAGCAGCAAGATAGAGAA |
| CLVd | 181 | T7_CLVd_F1 | TAATACGACTCACTATAGGGTTTTCACCCTTCCTTTC |
|  |  | CLVd_R1 | TGTTTCAGCTGGGATTACAC |
|  | 334 | T7_CLVd_F2 | TAATACGACTCACTATAGGGCGAGGGTGTTTAGCCCT |
|  |  | CLVd_R2 | GGAGACCAAGCAAGATAGAG |
| CCCVd | 3 | T7_CCCVdf_F1 | TAATACGACTCACTATAGGGGAAATCTACAGGGCAC |
|  |  | CCCVdf_R1 | AGAGGGGCTACAAAGGGAC |
|  | 148 | T7_CCCVdf_F2 | TAATACGACTCACTATAGGCCGCCCCTCCTCGAC |
|  |  | CCCVdf_R2 | GGGCGTCGAAGCTACGAAG |
| ASSVd | 1 | T7_ASSVd_F1 | TAATACGACTCACTATAGGTAAACACCGTGCGGTTCC |
|  |  | ASSVd_R1 | GGGAAACACCTATTGTGTTTTACCC |
|  | 157 | T7_ASSVd_F2 | TAATACGACTCACTATAGGGTTCGCCTACAAGAACG |
|  |  | ASSVd_R2 | ACAGCGGAACTGGTGCGAG |
| PBCVd | 109 | T7_PBCVd_F1 | TAATACGACTCACTATAGCGAGGGTGGAAGTTTACCG |
|  |  | PBCVd_R1 | CGCGAAGCCAAGCTGCTATA |
|  | 260 | T7_PBCVd_F2 | TAATACGACTCACTATACTAGAAGCCTGGGCGCTGGC |
|  |  | PBCVd_R2 | ACCCTTCGTCGACGACGAGC |
| CVd-OS | 1 | T7_CVd-OS_F1 | TAATACGACTCACTATAGGAGGAAACTCCGTGTGGTTCC |
|  |  | CVd-OS_R1 | GGGGAAACACCAATCGTG |
|  | 151 | T7_CVd-OS_F2 | TAATACGACTCACTATAGGACAGTAGAGCTCGTCTCTAC |
|  |  | CVd-OS_R2 | ATCGCTCGTCTCAGAGGCC |
| CbVd-1 | 82 | T7_CbVd-1_F1 | TAATACGACTCACTATAGGAATCCAGTGCCCACCGG |
|  |  | CbVd-1-2-3_R1 | GTTGCAGCGCTGCCAGGGAACCC |
|  | 193 | T7_CbVd-1_F2 | TAATACGACTCACTATAGGTTCGCTGACCCGGTTCG |
|  |  | CbVd-1_R2 | AGGACCTTTGCAATCGCTC |
| CbVd-2 | 108 | T7_CbVd-1-2_F1 | TAATACGACTCACTATAGGAATCCAGTGCCCACCGG |
|  |  | CbVd-1-2-3_R1 | GTTGCAGCGCTGCCAGGGAACCC |
|  | 214 | T7_CbVd-2_F2 | TAATACGACTCACTATAGGTCCCGGTTCGCTCTCC |
|  |  | CbVd-2_R2 | TTTGCAATCGCTCGCGGGG |
| CbVd-3 | 108 | T7_CbVd_F1 | TAATACGACTCACTATAGGAATTCAGGGCGTTGGCC |
|  |  | CbVd-1-2-3_R1 | GTTGCAGCGCTGCCAGGGAACCC |
|  | 262 | T7_CbVd-3_F2 | TAATACGACTCACTATAGGCGATTGCAAAGGATCCGG |
|  |  | CbVd-3_R2 | GCGTGGGAGCAGGTTGCTG |
